# Biomechanical role of cement augmentation in the vibration characteristics of the osteoporotic lumbar spine after lumbar interbody fusion

**DOI:** 10.1007/s10856-022-06671-6

**Published:** 2022-06-03

**Authors:** Qing-Dong Wang, Li-Xin Guo

**Affiliations:** 1 Department of Mechanical Engineering, Tsinghua University, Beijing, China; 2 School of Mechanical Engineering and Automation, Northeastern University, Shenyang, 110819 China

**Keywords:** Lumbar interbody fusion, Cement augmentation, Whole-body vibration, Complications, Fusion outcomes

## Abstract

Under whole body vibration, how the cement augmentation affects the vibration characteristic of the osteoporotic fusion lumbar spine, complications, and fusion outcomes is unclear. A L1-L5 lumbar spine finite element model was developed to simulate a transforaminal lumbar interbody fusion (TLIF) model with bilateral pedicle screws at L4-L5 level, a polymethylmethacrylate (PMMA) cement-augmented TLIF model (TLIF-PMMA) and an osteoporotic TLIF model. A 40 N sinusoidal vertical load at 5 Hz and a 400 N preload were utilized to simulate a vertical vibration of the human body and the physiological compression caused by muscle contraction and the weight of human body. The results showed that PMMA cement augmentation may produce a stiffer pedicle screw/rod construct and decrease the risk of adjacent segment disease, subsidence, and rod failure under whole-body vibration(WBV). Cement augmentation might restore the disc height and segmental lordosis and decrease the risk of poor outcomes, but it might also increase the risk of cage failure and prolong the period of lumbar fusion under WBV. The findings may provide new insights for performing lumbar interbody fusion in patients affected by osteoporosis of the lumbar spine.

**Figure:**
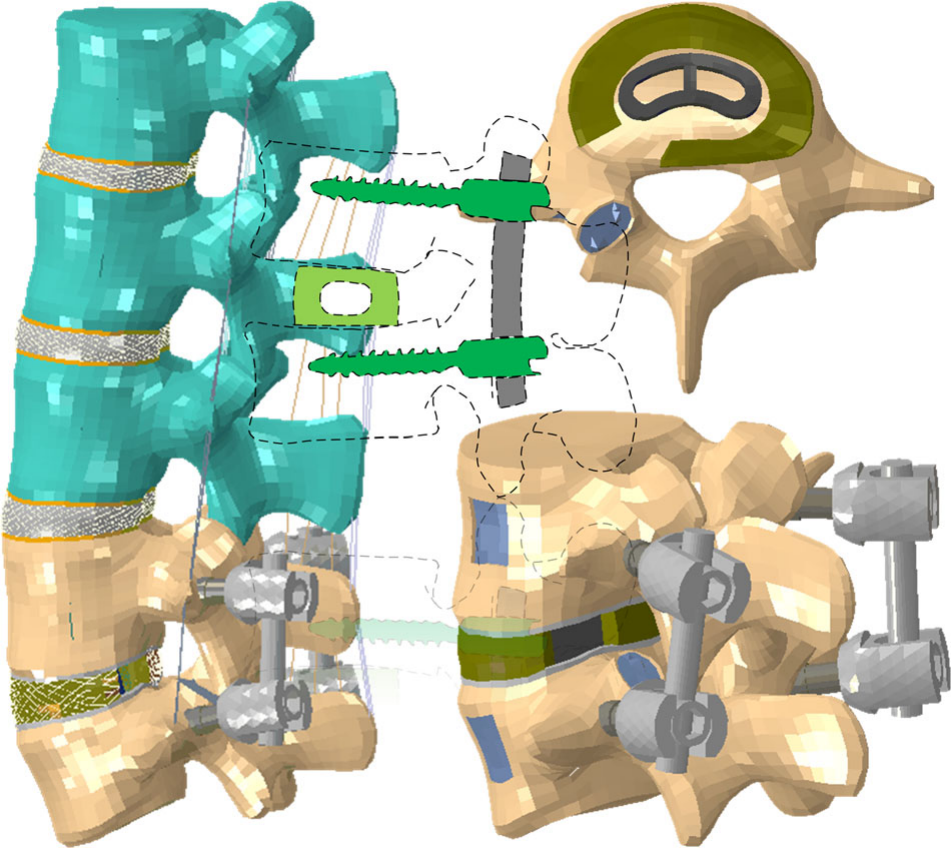
Graphical abstract

Graphical abstract

## Introduction

Lumbar interbody fusion combined with bilateral pedicle screw fixation has been used in facilitating arthrodesis and improving clinical outcomes for many years [1–4]. However, lumbar spine osteoporosis may result in fixation failure, nonunion, or other complications [5–10]. Most patients requiring lumbar interbody fusion are elderly with osteoporosis, and pedicle screw placement may lead to loss of bone density and osteoporosis in fused segments [11–14]. There are some methods to increase bone mineral density and enhance the fixation strength to improve the stability of osteoporotic lumbar spine. Among the various approaches, polymethylmethacrylate (PMMA) cement augmentation has yielded favorable results in treating patients with osteoporosis [15, 16]. Previous studies have evaluated the effects of PMMA cement augmentation on biomechanical properties of osteoporotic vertebral bone in fused segments. The experimental study by Liu et al. [17] reported that PMMA could significantly enhance screw stability, and there was a significant positive correlation between screw stability and volume of PMMA. A clinical study by Mo et al. compared the safety and efficiency of cement-augmented pedicle screw with a traditional pedicle screw technique applied to patients with osteoporotic spine. They found better fusion and lower pedicle screw loosening rates of the PMMA-augmented pedicle screw group in the single segment patients [18]. An experimental study by Tan et al. reported that cage-vertebra interface properties were improved when cement was used to augment vertebral and pedicle screws. They found that cement augmentation of pedicle screws might reduce interbody device subsidence [19]. Similarly, there are also a lot of numerical results from the finite element analysis. The finite element analysis by Ferris et al. demonstrated that placement of cement influenced failure load and toggle, with distal placement having the largest increase in failure load and decrease in cephalad-caudad toggle [20]. Polikeit et al. investigated the effect of cement augmentation on an osteoporotic fusion lumbar spinal unit by using finite element analysis. They found that cement augmentation improved the strength of osteoporotic vertebrae, but increased endplate bulge and the load in the adjacent segments [21]. There are many valuable experimental and numerical results about the effects of PMMA cement augmentation on biomechanical properties of the osteoporotic fusion lumbar spine, as well as the fusion outcomes and complications. However, a few studies focused on the effects of cement augmentation on the osteoporotic vertebral bone in fused segments under WBV.

Long-time whole-body vibration might lead to low back pain during driving a car or taking public transportation [22, 23]. A lot of experimental, clinical and numerical results attributed this to the fact that compared to static loads, vibration loads might result in increases in stress, intradiscal pressure(IDP) and disc degeneration in the lumbar spine [24, 25]. In daily life, people usually suffer from influence of WBV, such as taking a bus or driving a car, which might cause damage to the lumbar spine, especially to the osteoporosis elderly undergoing lumbar interbody fusion. Therefore, the role of cement augmentation in dynamic behaviors of the osteoporotic spinal segment under vibration has been widely concerned recently. Finite element analysis by Su et al. examined the influence of cement augmentation on the dynamics of pathologic and adjacent vertebrae under vibration conditions [26]. Bostelmann et al. assessed the fixation effect of percutaneous cement application and investigated pedicle screw loosening under physiological cyclic craniocaudal loading [27]. The purpose of this study is to investigate the influence of cement augmentation on the vibration characteristics of osteoporotic vertebral bone in fused segments, including the effect on adjacent segments and the fused segment, especially regarding fusion outcomes and complications such as adjacent segment diseases (ASD), pedicle screw fixation failure, and subsidence.

## Methods

### FE modeling and materials

**Fig. 1:**
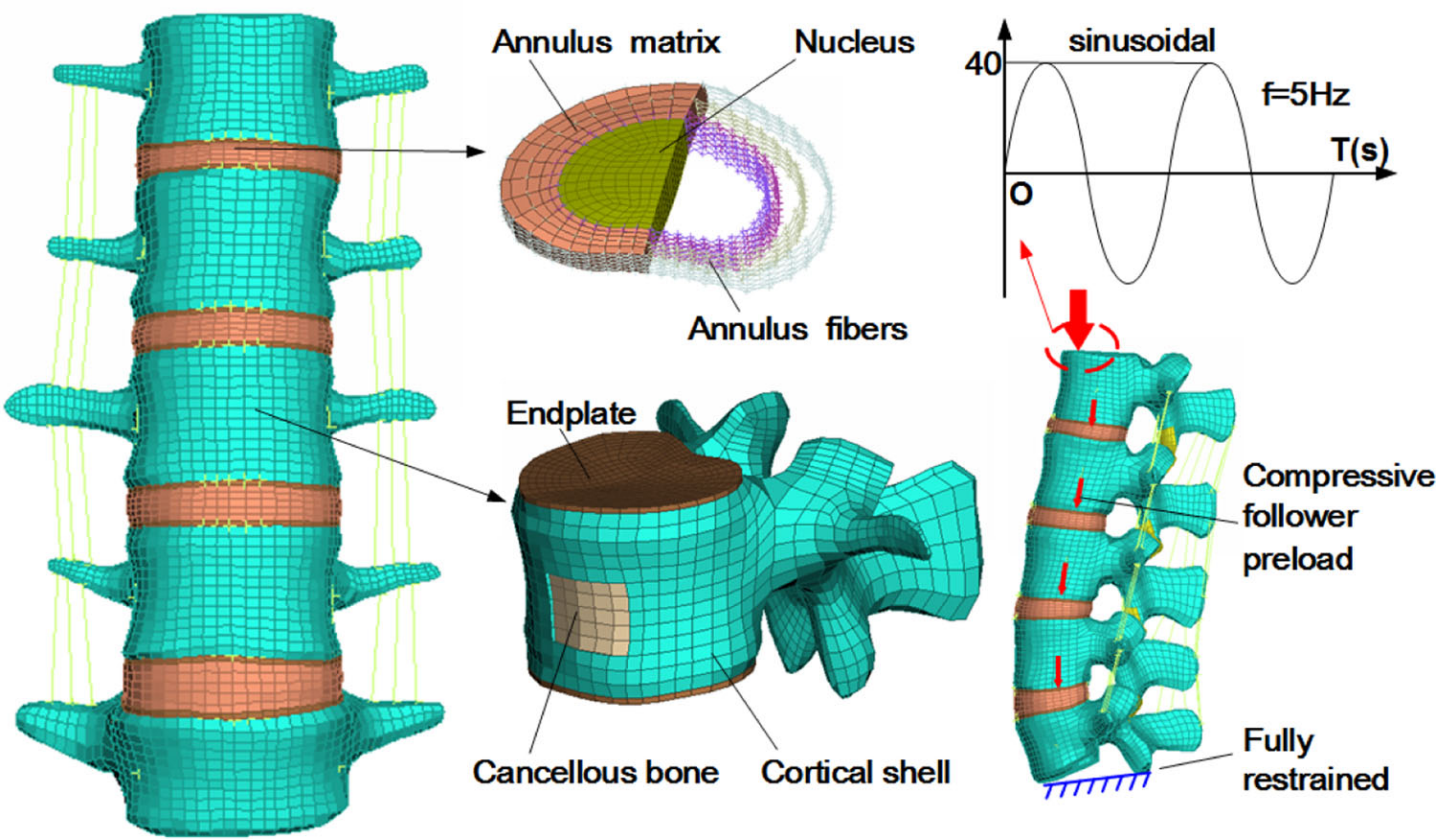
Finite element model of the intact L1-L5 of human lumbar spine

A previously validated three-dimensional nonlinear FE model of an intact L1-L5 lumbar spine was used in this study [28]. The intact model was composed of the vertebral body, endplate, intervertebral disc, and various ligaments such as anterior longitudinal, posterior longitudinal, capsular, intertransverse, interspinous, supraspinous, and flavum ligaments. The intervertebral disc consisted of annulus fibrosus, annulus ground substance and nucleus pulposus, as shown in Fig. 1. The elastic modulus of annulus fibrosus decreased from the outside to the inside.

**Fig. 2:**
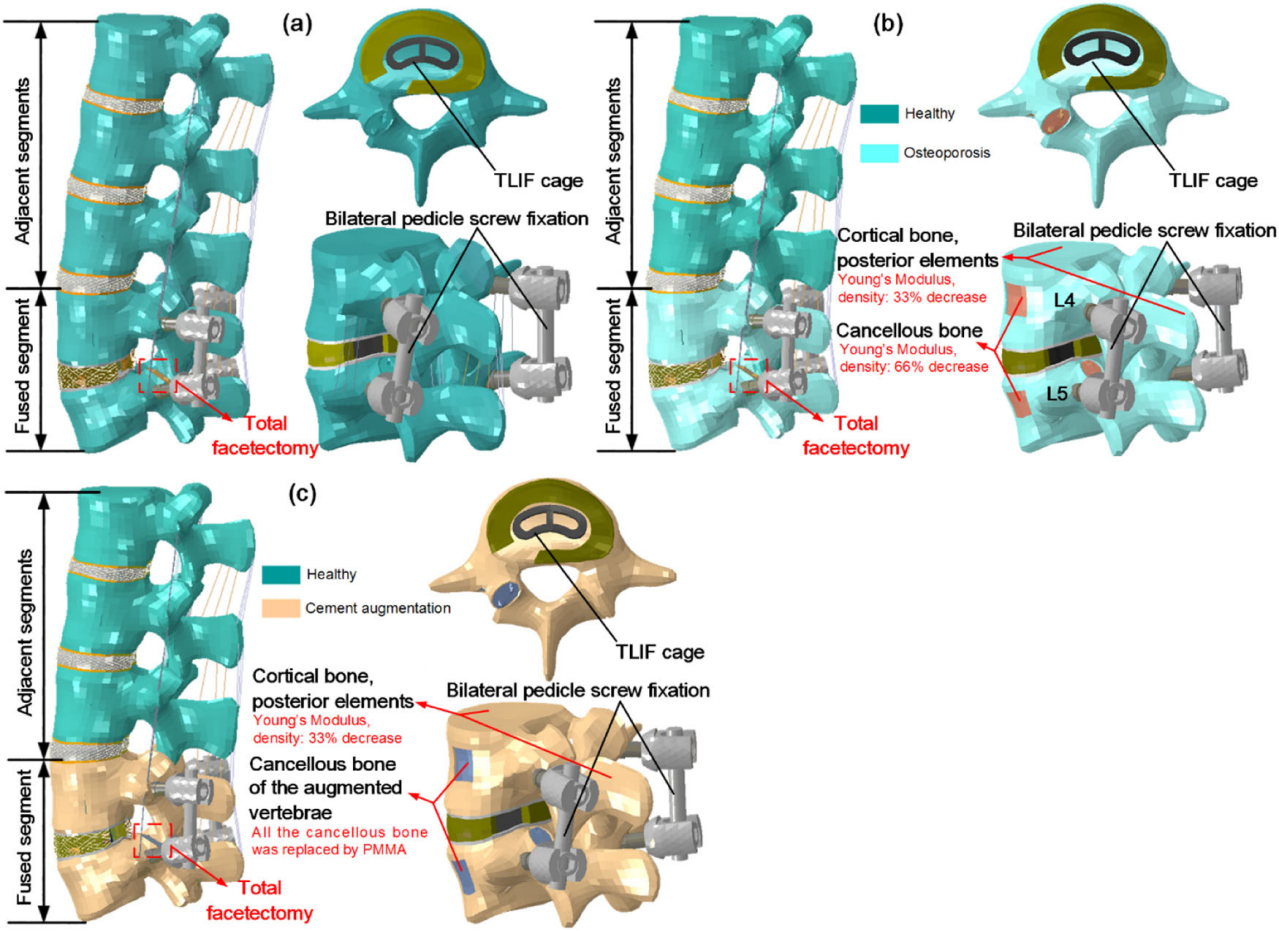
Finite element models for different conditions. **a** TLIF model **b** TLIF model with osteoporotic L4, L5 lumbar vertebrae **c** PMMA cement augmented TLIF model

A lot of investigations indicated that the prevalence of spinal diseases at L4-L5 was the greatest among lumbar intervertebral discs [29, 30], and transforaminal lumbar interbody fusion (TLIF) might provide better biomechanical stability and decrease the risk of complications [31, 32]. Therefore, an intact L1-L5 model was modified to simulate TLIF at the L4-L5 level. To simulate TLIF, the nucleus pulposus, partial lamina, partial annulus ground substance, fibrosus, and unilateral superior articular process were removed at the L4-L5 level. A cage (length 28 mm, width 12 mm, height 10 mm) was inserted into the disc space by the oblique approach through the annulus incision (Fig. 2). The endplate-cage interfaces, bone-screw interfaces and pedicle screw-rod interfaces were assumed to be bonded. The material properties were assumed to be homogeneous and isotropic, the corresponding data [33–37] were given in Table 1.

**Table 1:** Material properties of the finite element model

Component	Element type	Young’s modulus (MPa)	Poisson’s ratio	Density (e-6 Kg/mm^3^)	Cross-sectional area (mm^2^)
Bone					
Cancellous bone	C3D4	100(osteoporosis:34)	0.2	1.1(osteoporosis:0.37)	
Cortical bone	C3D8	12000(osteoporosis:8040)	0.3	1.7(osteoporosis:1.14)	
Posterior bone	C3D4	3500(osteoporosis:2345)	0.25	1.4(osteoporosis:0.94)	
Endplate	C3D8	500	0.25	1.2	
Intervertebral disc					
Nucleus pulposus	C3D8	1	0.49	1.02	
Annulus ground substance	C3D8	4.2	0.45	1.05	
Annulus fibers	T3D2	357–550	0.3	1.0	
Ligaments					
Anterior longitudinal	T3D2	7.8(<12.0%) 20.0(>12.0%)		1.0	63.7
Posterior longitudinal	T3D2	10.0 (<11.0%) 20.0 (>11.0%)		1.0	20
Capsular	T3D2	7.5(<25.0%) 32.9 (>25.0%)		1.0	30
Intertransverse	T3D2	10.0(<18.0%) 58.7 (>18.0%)		1.0	1.8
Interspinous	T3D2	10.0 (<14.0%) 11.6 (>14.0%)		1.0	40
Supraspinous	T3D2	8.0 (<20.0%) 15 (>20.0%)		1.0	30
Ligamentum flavum	T3D2	15.0 (<6.2%) 19.5 (>6.2%)		1.0	40
Implants					
TLIF cage(PEEK)	C3D8	3600	0.25	1.32	
Screw and rod(Ti)	C3D4	110,000	0.28	4.5	

Lumbar vertebral bodies fixed by bilateral pedicle screws were prone to osteoporosis. In this study, the L4 and L5 vertebrae were assumed to be osteoporotic, and other vertebral bodies were healthy in the “TLIF model with osteoporosis” (TLIF-OST). To simulate the TLIF with osteoporosis, the elastic moduli values of the osteoporosis based on calculations relating bone mineral density measurements in healthy and osteoporotic bones were collected [21, 38]. The fusion model with osteoporosis (Fig. 2) was defined as follows. The Young’s Modulus and density of the cancellous bone in L4 and L5 were reduced by 66%, and those of cortical bone and posterior elements in L4 and L5 were decreased by 33% [26, 39]. To simulate the PMMA cement augmented TLIF model (TLIF-PMMA), all the cancellous bone of the augmented L4/5 vertebrae was replaced by PMMA (Young’s Modulus: 3000 MPa, Poisson’s Ratio: 0.41), and other parameters were the same as the TLIF-OST model [40].

### Boundary and loading conditions

In this study, Abaqus 6.14(Dassault Systemes Simulia Corp) was used to analyze the effect of PMMA cement augmentation on the osteoporotic vertebral bone in fused segments. For boundary conditions, the lower surface of L5 vertebral body was fixed in all directions throughout the simulation process. A 400 N compression preload and a 40 N sinusoidal vertical load at 5 Hz were applied to the models to simulate the physiological compression load of the whole lumbar spine caused by muscle contraction and the weight of human body and vibration load of human body in many vehicle transportations, respectively [25, 41–43]. A 40 kg mass point was designated on the top of L1 to simulate the effect of human upper body mass on the lumbar spine [44–46].

## Results

The numerical results about dynamic characteristics of the models including the vibration amplitude and maximum value of von Mises stress in L4/L5 endplates, cage, pedicle screw, AGS and IDP were collected. Some significant indexes related to fusion outcomes and complications, such as segmental lordosis, disc height and compressive stress in L4/5 endplates were also analyzed. The results were periodic, and a representative period of 0–0.8 s was chosen from the entire vibration process (2 s) in this study.

### Effect of cement augmentation on adjacent segments

**Fig. 3:**
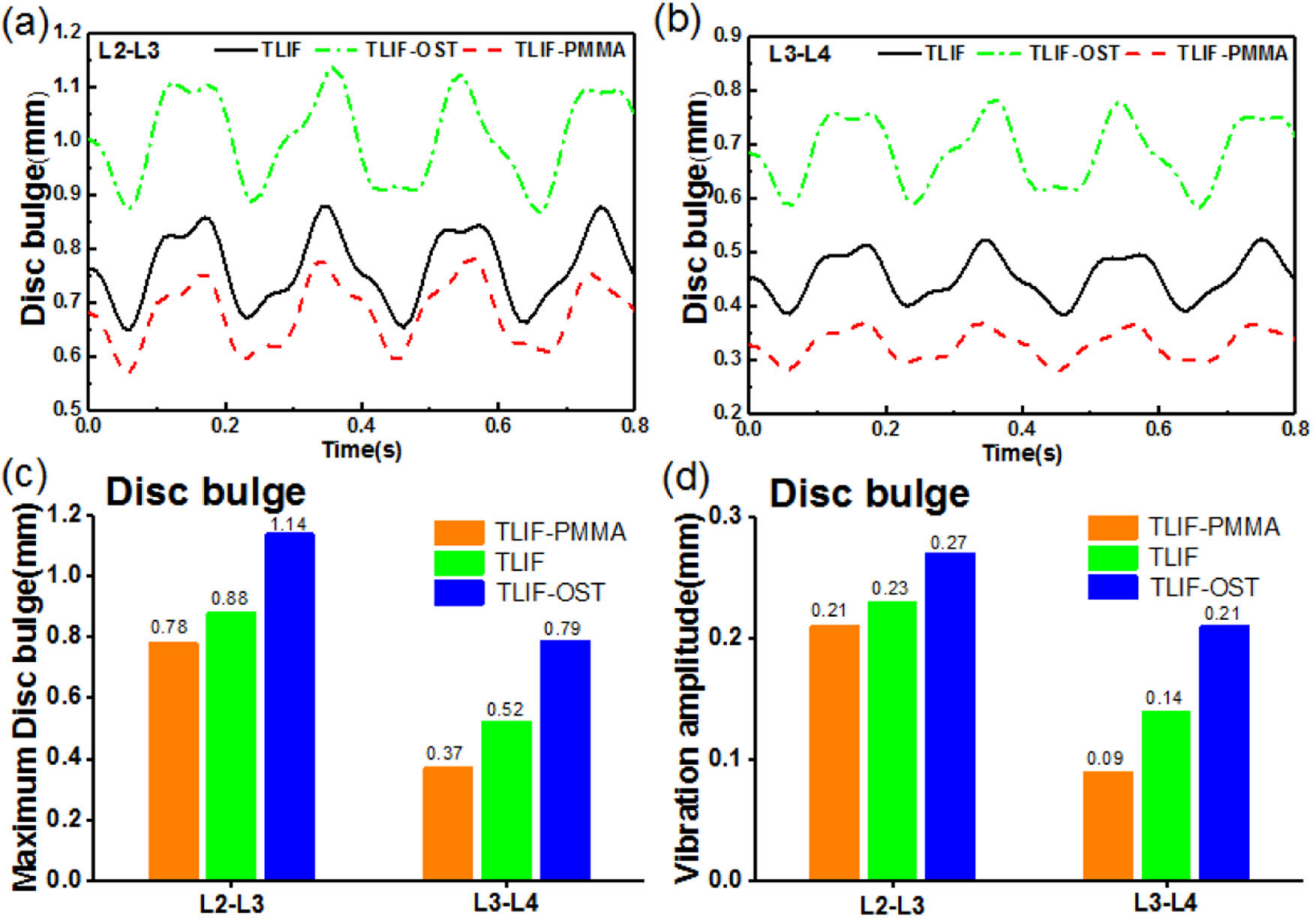
The dynamic response at the adjacent segments for fusion models. **a** L2-L3 disc bulge **b** L3-L4 disc bulge **c** the maximum values of L2-L3 and L3-L4 disc bulge **d** vibration amplitudes of L2-L3 and L3-L4 disc bulge

**Fig. 4:**
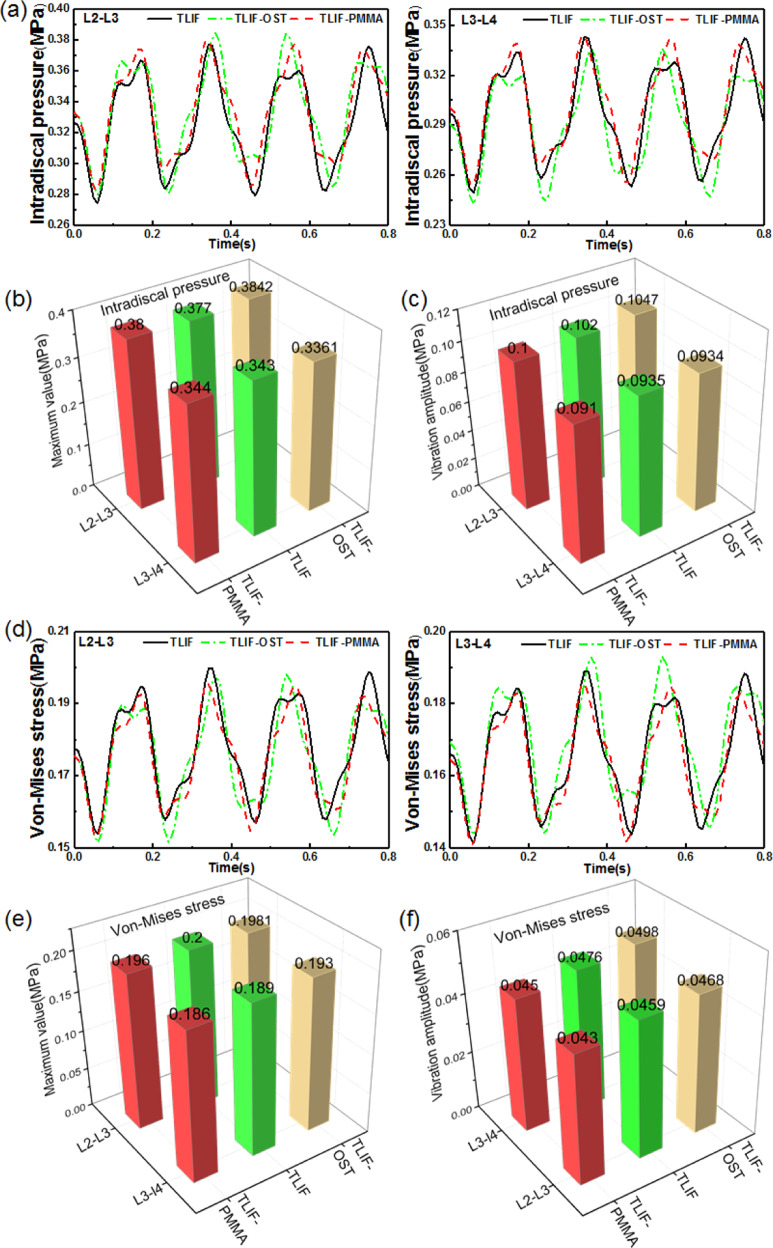
The dynamic response at the adjacent segments (L2-L3 and L3-L4 levels) for fusion models. **a** IDP, **b** the maximum values of IDP, **c** vibration amplitudes of IDP, **d** Von-Mises stress in the AGS, **e** the maximum values of the stress in AGS, **f** vibration amplitudes of the stress in AGS

At the adjacent segments, there are some indexes related to ASD such as disc bulge, IDP, and von Mises stress in the annulus fibrosus. The disc bulge was defined as the lateral deformation of the annulus fibrosus, and the IDP and von Mises stress were assumed to be the average stress in the elements. It was found that TLIF-PMMA exhibited the smallest dynamic response in the disc bulge of L2/3 and L3/4 levels among the three models in Fig. 3. For example, the maximum values (vibration amplitudes) of L2/3 and L3/4 disc bulge were 0.78(0.21) mm, 0.37(0.09) mm for TLIF-PMMA, 0.88(0.23) mm, 0.52(0.14) mm for TLIF, 1.14(0.27) mm, and 0.79(0.21) mm for TLIF-OST. It was observed that no matter the maximum values or vibration amplitudes in Fig. 4, there was no obvious difference in IDP and stress in AGS of L2/3 and L3/4 levels among TLIF, TLIF-OST and TLIF-PMMA models.

### Effect of cement augmentation on adjacent segments

**Fig. 5:**
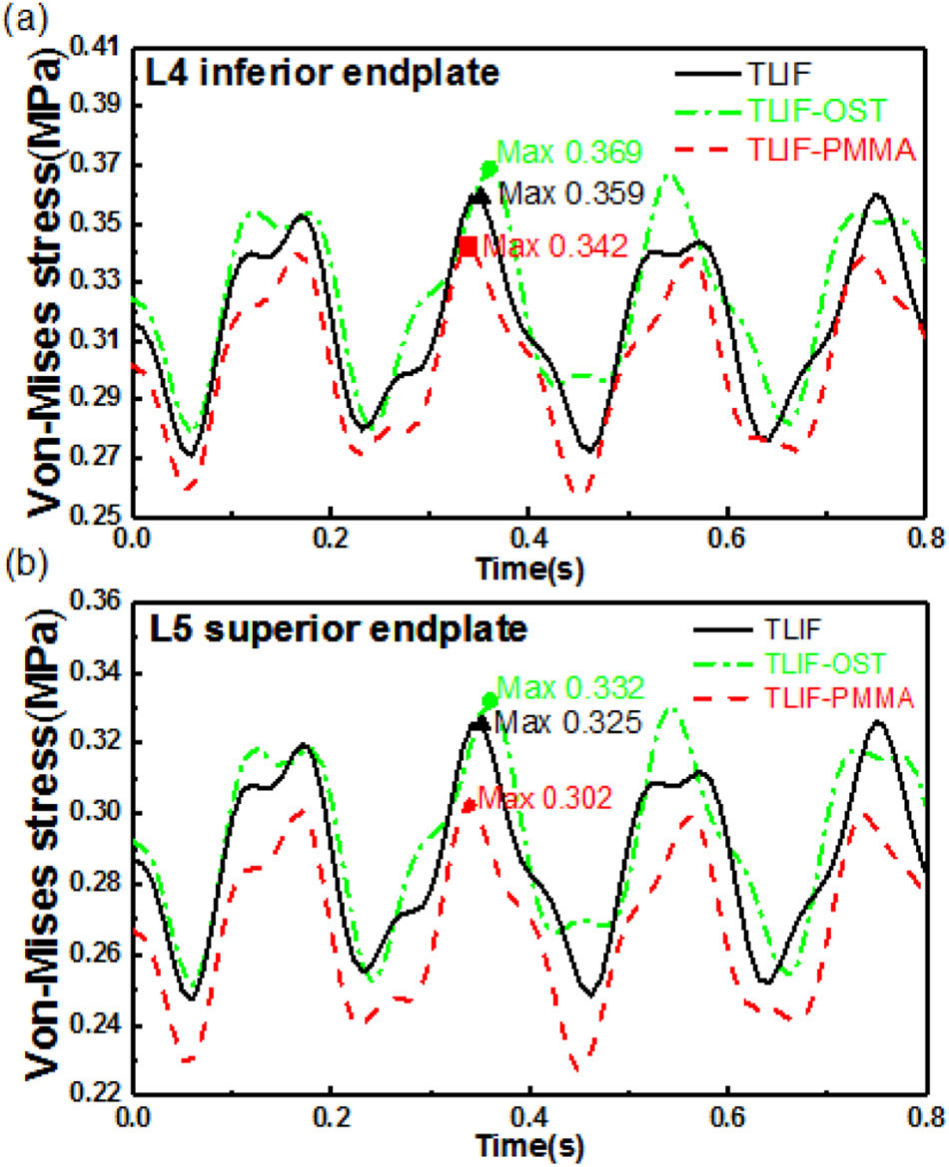
Dynamic response of von Mises stress. **a** L4 inferior endplate and **b** L5 superior endplate for the fusion models

**Fig. 6:**
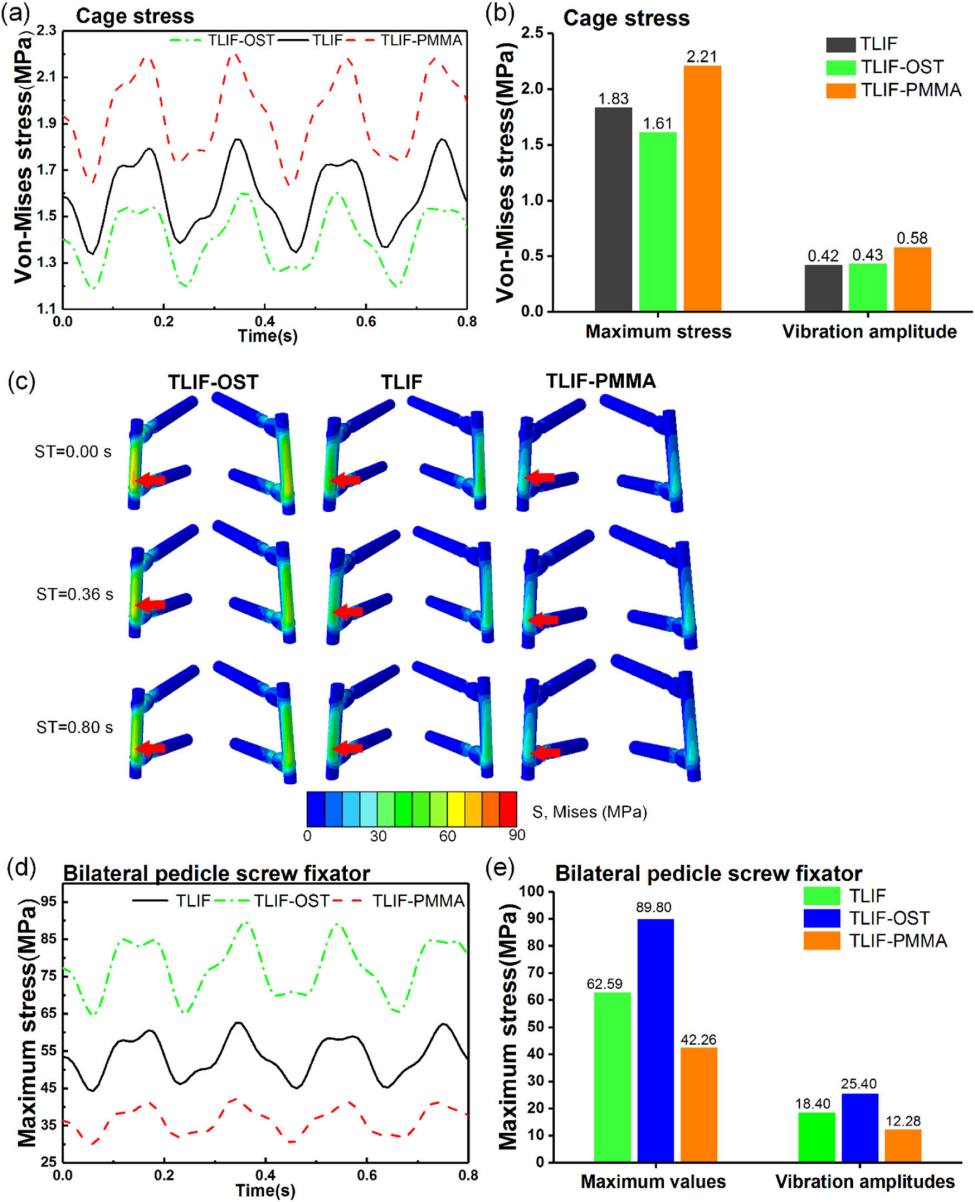
Dynamic response of the maximum stress in the cage and bilateral pedicle screw for the fusion models. **a** the von Mises stress in the cage, **b** the maximum values and vibration amplitudes of the von Mises stress in the cage, **c** von Mises stress distributions of the bilateral pedicle screw in the TLIF, TLIF-OST, and TLIF-PMMA models when the step time (ST) was 0.00, 0.36, and 0.80 s. The red arrow shows the location of the maximum stress in pedicle screw, **d** the maximum stress in pedicle screw, **d** the maximum values and vibration amplitudes of the maximum stress in pedicle screw

For the fused L4-L5 level, the stresses in endplates, cage and pedicle screw were pertinent with complications such as subsidence, cage and fusion failure. The stresses in endplates and cage were the average stress in the elements. As illustrated in Fig. 5, the TLIF-PMMA decreased the maximum values of the L4 inferior and L5 superior endplates compared with TLIF-OST and TLIF models. For example, the maximum values of the L4 inferior and L5 superior endplates were 0.342 MPa, 0.302 MPa for TLIF-PMMA, 0.359 MPa, 0.325 MPa for TLIF, and 0.369 MPa, 0.332 MPa for TLIF-OST, respectively. It was found, in Fig. 6a, b, that TLIF-PMMA generated the greatest cage stress among the TLIF, TLIF-OST, and TLIF-PMMA models. The maximum cage stresses of TLIF-PMMA, TLIF-OST, and TLIF models were 2.21 MPa, 1.61 MPa and 1.83 MPa, respectively.

The von Mises stress distribution of the bilateral pedicle screw in TLIF-PMMA, TLIF-OST and TLIF models (Fig. 6c) indicated that TLIF-PMMA reduced the high stress concentration intensity compared with other models, and the high stress concentration regions were mainly at the rod and the neck of pedicle screw. It was found (Fig. 6d,e) that the maximum stresses in the pedicle screw or the rod of TLIF-PMMA were smaller than those of TLIF-OST and TLIF models. The maximum values (vibration amplitudes) of the maximum stress in the pedicle screw were 42.26(12.28)MPa for TLIF-PMMA, 62.59(18.40) MPa for TLIF, and 89.90(25.40) MPa for TLIF-OST.

### Effect of cement augmentation on disc height, segmental lordosis and compressive stress at the fused level

**Fig. 7:**
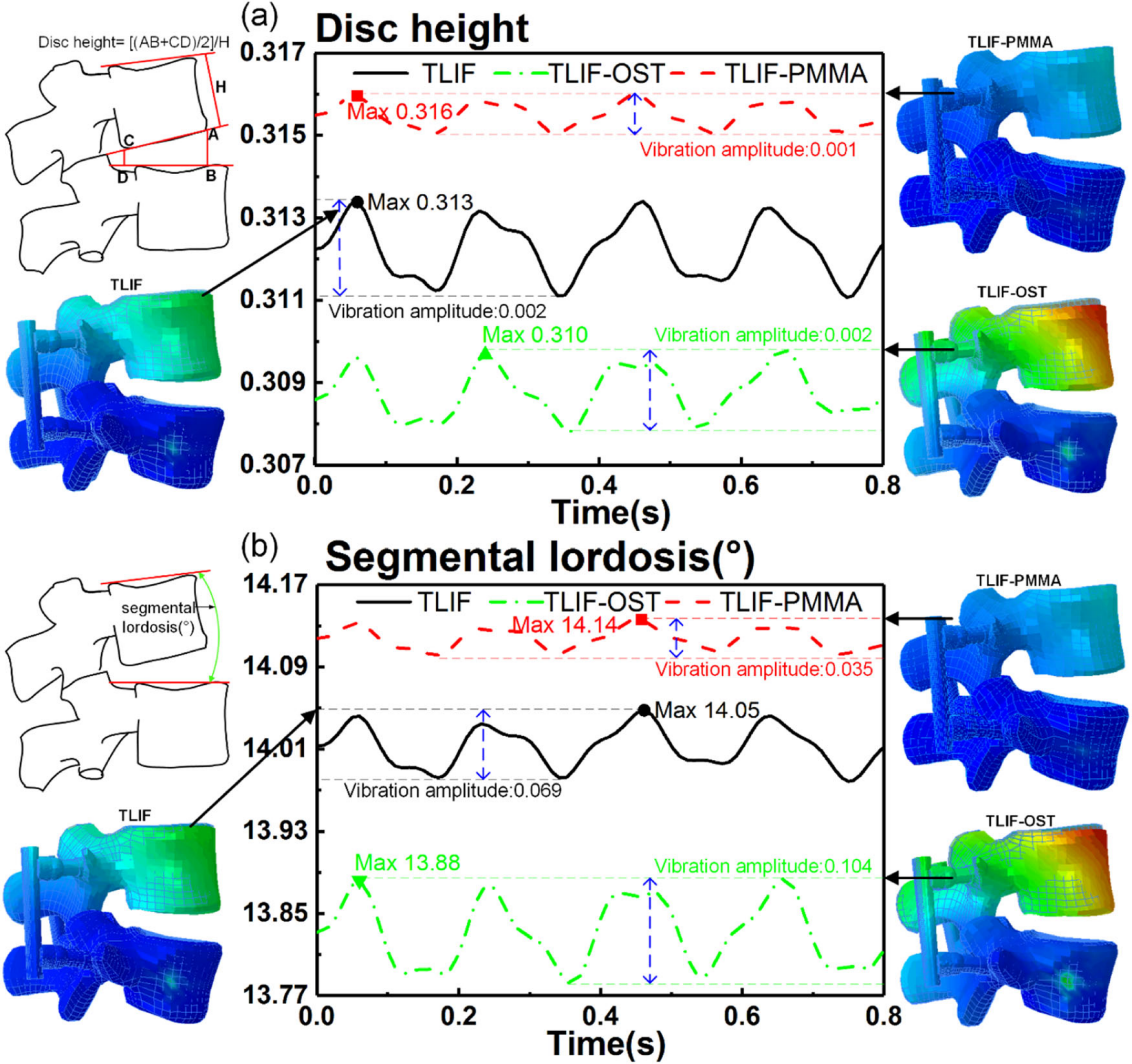
The dynamic response of **a** disc height and **b** segmental lordosis at L4-L5 fused level for TLIF, TLIF-OST and TLIF-PMMA models. Segmental lordosis: the angle represented by green arc

**Fig. 8:**
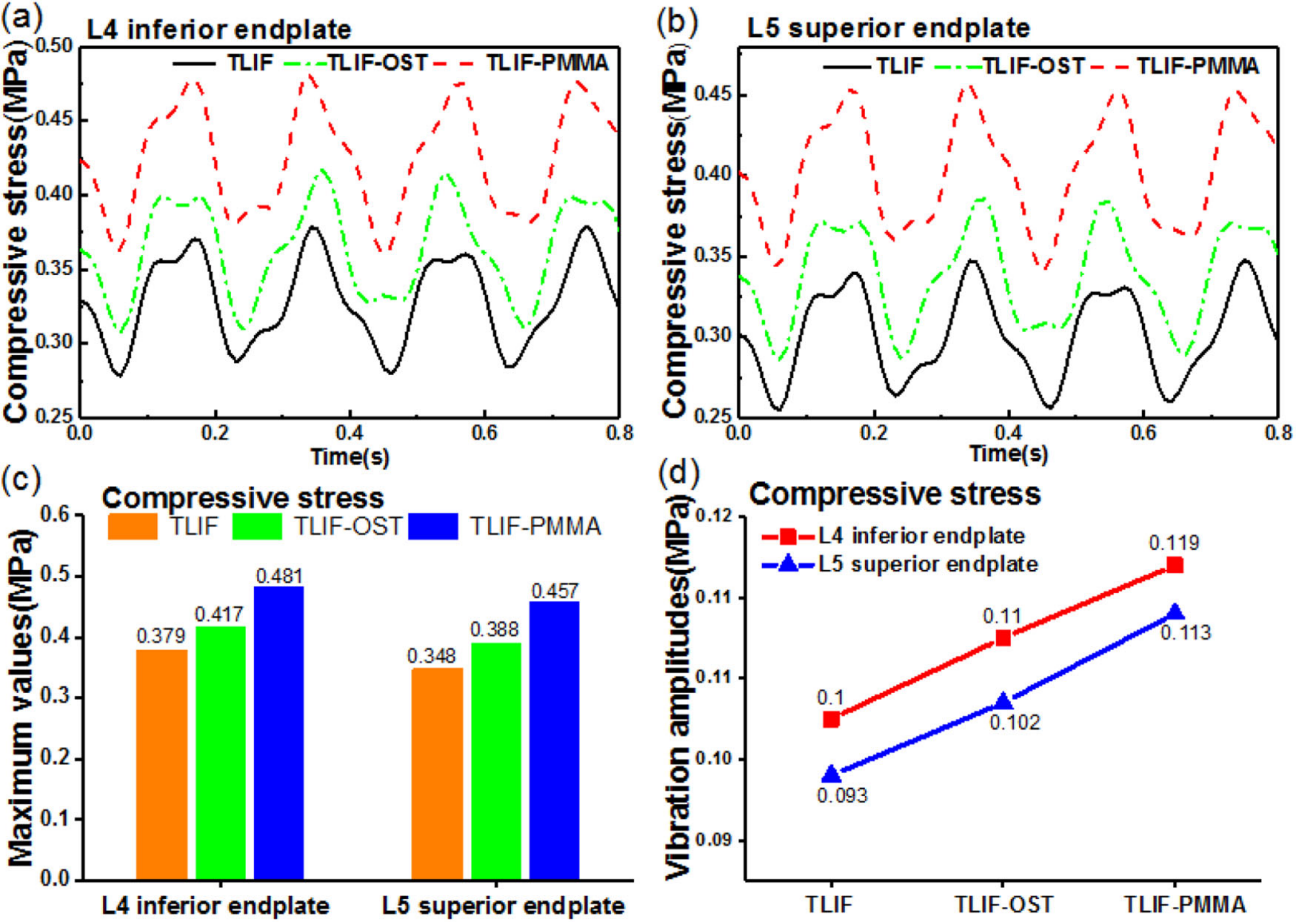
The dynamic response of the compressive stress at the interface between cages and endplates for TLIF, TLIF-OST, and TLIF-PMMA models. The compressive stress in **a** the interface between cage and L4 inferior endplate, **b** the interface between cage and L5 superior endplate, **c** the maximum values of the compressive stress and **d** vibration amplitudes of the compressive stress

The disc height, segmental lordosis and compressive stress at the interfaces between the cage and endplates were closely related to complications and outcomes. A disc height measurement method reported by Drain et al. was adopted in this study [47]. The segmental lordosis was measured only at the fused level (L4-L5). The compressive stress was assumed to be the average stress of elements. As illustrated in Fig. 7 the disc height and segmental lordosis of TLIF-PMMA were greater than those of TLIF-OST and TLIF models during the entire simulation process. The maximum disc height (vibration amplitude) of TLIF-PMMA, TLIF and TLIF-OST models were 0.316(0.001) mm, 0.313(0.002) mm, and 0.310(0.002)mm, respectively. The dynamic responses (the maximum values and vibration amplitudes) in segmental lordosis were 14.14°(0.035) for TLIF-PMMA, 14.05°(0.069) for TLIF, 13.88°(0.104) for TLIF-OST. As illustrated in Fig. 8, TLIF-PMMA model generated greater compressive stresses in the L4 inferior and L5 superior endplates compared with TLIF-OST and TLIF models. The maximum compressive stresses (vibration amplitude) in the L4 inferior and L5 superior endplates were 0.481(0.119) MPa and 0.457(0.113) MPa for TLIF-PMMA, 0.417(0.110) MPa and 0.388(0.102) MPa for TLIF-OST, 0.379(0.100) MPa and 0.348(0.093) MPa for TLIF.

## Discussion

Cement augmentation in treating the osteoporotic vertebral bone in fused segments under static load has been widely investigated, but a few studies dealt with WBV. Therefore, this study evaluated the effects of cement augmentation on the vibration characteristics of osteoporotic vertebral bone in fused segments, including the influence on adjacent segments and fused segment, to investigate the relationship between cement augmentation, fusion outcomes and complications under WBV. In this study, an intact L1-L5 lumbar spine model was developed to simulate TLIF-PMMA, TLIF-OST, and TLIF models with bilateral pedicle screw fixation at L4-L5 level. For the TLIF-PMMA model, all the cancellous bone of L4/5 vertebrae was replaced by PMMA. The Young’s Modulus and density of L4 and L5 vertebral bodies were reduced in the TLIF-OST model. Most patients undergoing lumbar fusion surgery were elderly with osteoporosis. The vibration load of this study was a 40 N, 5 Hz vertical sinusoidal vibration load, similar to other studies [25, 48, 49].

For the adjacent segments, there was no difference among the TLIF-PMMA, TLIF-OST, and TLIF models in the dynamic responses of IDP and stress in AGS. Furthermore, the TLIF-PMMA decreased the maximum values and vibration amplitudes of L2/3 and L3/4 disc bulge compared with the TLIF-OST and TLIF models. The findings imply that PMMA cement augmentation may give no increase of incidence of adjacent segment diseases, and it may leave the adjacent segments in a more stable condition and decrease the risk of ASD. A clinical study by Kim et al. reported the same trend that PMMA augmentation did not increase the nonunion rate and incidence of ASD [50].

For the fused segment (L4-L5 level), the stresses in the cage, pedicle screw, and endplates are related to cage failure, fixation failure and subsidence. The TLIF-PMMA decreased the maximum values of stress in L4/5 endplates compared with TLIF-OST and TLIF models. Based on this result, we believe that PMMA cement augmentation may decrease the risk of cage subsidence under WBV. A similar conclusion reported by Park et al. and Kim et al. that PMMA cement augmentation in vertebral bodies could resist cage subsistence [51].

**Fig. 9:**
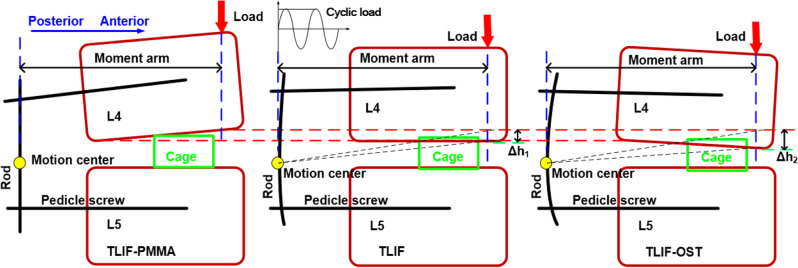
The schematic diagrams to illustrate the load-transferring mechanism of the TLIF-PMMA, TLIF, and TLIF-OST models at the fused (L4-L5) level

In this study, the TLIF-PMMA model generated the maximum value and vibration amplitude of stress in the cage than TLIF-OST and TLIF models. This result was consistent with the research by Polikeit et al. who reported that the greater Young’s Modulus of cancellous bone, the more the stress was concentrated underneath the cage, while the remaining regions were unloaded [52]. In this study, the results showed high stress concentration regions were at the rod and neck of pedicle screw, and TLIF-PMMA could decrease the high stress intensity compared with TLIF-OST and TLIF models. Some researchers predicted the same trend about the stress concentration region in rods and pedicle screws [53, 54]. The TLIF-PMMA model decreased the maximum values (vibration amplitudes) of stress in the fixator compared with other models. The mechanical analysis of the cage and fixator was as follows. After instrumentation, the load was shared by the cage, vertebral body and bilateral pedicle screw fixator, as shown in Fig. 9. Due to osteoporosis decreasing the strength of vertebrae, the load shared by the fixator was increased in the fused lumbar spine with osteoporosis. When the load was on the top of L4, there was the height difference (Δh_2_) between the disc height of L4-L5 in TLIF-PMMA model and TLIF-OST model, as shown in Fig. 9. The motion center (yellow point) was the region where the rod (fixator) was more prone to failure in TLIF-OST model. PMMA cement augmentation restores the strength of the fused vertebrae. The load shared by the cage was increased in the TLIF-PMMA model. Therefore, the cage stress of TLIF-PMMA model was greater than that of TLIF-OST model. Based on above results, we infer that PMMA cement augmentation might provide a stiffer pedicle screw/rod construct and decrease the risk of rod failure (fixator failure), but increase the risk of cage failure under WBV.

Many studies have shown that loss of lordosis and disc height may lead to some complications such as poor fusion outcomes, ASD, disc degeneration, etc [55–57], the compressive stress of the interface between cage and endplates might inhibit the growth of vertebral cells and result in poor outcomes [58]. After cement augmentation, the TLIF-PMMA model restored the disc height and segmental lordosis of the fused segment (L4-L5 level) compared with TLIF-OST model. The vibration amplitudes of the disc height and segmental lordosis in TLIF-PMMA model were the smallest among TLIF-OST, TLIF and TLIF-PMMA. These imply that cement augmentation might restore the disc height and segmental lordosis and decrease the risk of adjacent segment and poor outcomes under WBV. Mo et al. [18] came to the similar conclusion that the cement augmented technique was effective and safe in the osteoporotic spine with lumbar degenerative diseases, with better fusion and less screw failure incidence. The TLIF-PMMA model exhibited larger compressive stress (vibration amplitude) of the interfaces between the endplates and cage than TLIF-OST and TLIF models during the entire vibration process. The stress-growth curve of vertebral cells indicated that the greater compressive stress, the more inhibition of the vertebral cell growth [59]. A lot of studies demonstrated that cement augmentation was capable to improve the stability of instrumentation and achieve satisfactory fusion with a rate from 92.5 to 100% in poor spinal bone [60–63]. All in all, the fusion outcomes were affected by a lot of factors such as the stability of instrumentation (stable environment) and a suitable growth environment for vertebral cells. The poor stability of instrumentation might directly lead to poor fusion outcomes, but the unsuitable growth environment (compressive stress) only increased the period of lumbar fusion. Many researches have demonstrated that cement augmentation might increase the stability of instrumentation, including this study. Therefore, this finding suggests that cement augmentation may prolong the period of lumbar fusion under WBV.

There are a few potential limitations inherent in this study. The material properties, including viscoelastic characteristics of intervertebral disc, non-linear behavior of spinal ligaments, degenerative changes caused by osteophytes, and the possible time-varying changes in disc properties were neglected, and the replacing the whole vertebral cancellous bone with PMMA in the model was an over-approximation, as only part of it was replaced with cement during vertebroplasty/kyphoplasty in the real clinical setting. In addition, a 400 N follower preload applied to the model could not entirely replace the complex contribution of muscles to the spine. However, in fact, these simplifications did not make a large influence on the results of this study.

## Conclusions

In this study, we investigated the effects of cement augmentation on the vibration characteristics of osteoporotic fusion lumbar spine to analyze the relationship between cement augmentation, fusion outcomes and complications under WBV. The results showed that PMMA cement augmentation might leave the adjacent segments in a more stable condition, and it might provide a stiffer pedicle screw/rod construct and decrease the risk of ASD, subsidence and rod/screw failure (fixator failure). Cement augmentation may restore the disc height and segmental lordosis and decrease the risk of poor outcomes under WBV, but it may increase the risk of cage failure and prolong the period of lumbar fusion. The findings may help us understand the effect of cement augmentation on the vibration characteristics of osteoporotic lumbar spine with cement augmentation.
